# Disinfection of the air and surfaces in the dental clinic using hydroxyl radical (OH-) based technology: A systematic review

**DOI:** 10.4317/jced.60461

**Published:** 2023-06-01

**Authors:** Anais Paños-Crespo, Bassel Traboulsi-Garet, Maria-Ángeles Sánchez-Garcés, Cosme Gay-Escoda

**Affiliations:** 1 DDS. Master in Oral Surgery and Buccofacial Implantology. Faculty of Medicine and Dentistry, University of Barcelona. Barcelona (Spain); 2 MD, DDS, MS, PhD, EBOS. Associate Professor of Oral Surgery. Faculty of Medicine and Dentistry, University of Barcelona. Researcher of the IDIBELL Institute. Barcelona (Spain); 3 MD, DDS, MS, PhD, EBOS, OMFS. Chairman of Oral and Maxillofacial Surgery. Faculty of Medicine and Dentistry, University of Barcelona. Director of the Master in Oral Surgery and Buccofacial Implantology (EFHRE International University / FUCSO). Coordinator / Researcher of the IDIBELL Institute. Head of the Department of Oral Surgery, Buccofacial Implantology and Maxillofacial Surgery. Teknon Medical Center. Barcelona (Spain)

## Abstract

**Background:**

A systematic review was carried out to compare the disinfectant capacity of hydroxyl radicals (OH-) versus other products commonly used for disinfecting the air and surfaces.

**Material and Methods:**

A literature search was made of the Cochrane Library, PubMed (MEDLINE) and Scopus databases. “In vitro” studies evaluating disinfection methods applicable to several surfaces and room air were included in the search. The search was carried out in April 2022, with no restrictions in terms of language or publication date.

**Results:**

Of the 308 articles identified from the initial search, 8 were included for the quantitative analysis. All publications corresponded to experimental “in vitro” studies. Seven of them evaluated biocidal action against bacteria, and only two assessed activity against viral loads. The generation of contaminants secondary to application of the disinfectants was only analyzed in one of the studies, with the conclusion that the production of peroxyl radicals (RO2) derived from the oxidation of volatile organic compounds (VOCs) is greater when chemical surface disinfectants are used versus air disinfection systems.

**Conclusions:**

The disinfection capacities of the currently available methods are similar, and none of them are able to replace the use of additional physical protection measures.

** Key words:**Disinfection methods, hydroxyl radical, environment, surfaces, dentistry.

## Introduction

Disinfection is a fundamental procedure characterized by the elimination of pathogenic microorganisms from surfaces, the air and inert objects (1). In contrast to sterilization, however, disinfection does not destroy spores (2).

Since the World Health Organization (WHO) declared the state of alarm due to the pandemic caused by the SARS-CoV-2 virus in 2020, concern about the quality of room air has increased exponentially, and thus also interest in new technologies capable of reducing environmental contamination, with the aim of avoiding the spread of cross-infections (3).

The disinfection of both room air and surfaces is of great importance in dental clinics, where routine practice is characterized by the important generation of aerosols, and where contact between the professional and the patient is direct (4). Accordingly, dental professionals play a key role in preventing the transmission of bacterial, viral and fungal infections (5).

The incubation period of the infections ranges between 2-14 days. During this prodromal phase, patients remain asymptomatic; all patients therefore should be regarded as potential sources of risk (6). The environment moreover plays an important role in the chain of transmission, since many microbiological agents are able to survive for prolonged periods of time on inert surfaces (7).

An adult inhales an average of 700 liters of air per hour, and patients undergoing dental treatment moreover typically experience hyperventilation (4). On the other hand, the oral cavity constitutes an ecological niche for over 700 species of bacteria – many of which are resistant to antimicrobials (8). All this implies that the bidirectional spread of infections is very high (4).

Cross-infection may occur through indirect contact with surfaces on which fomites and aerosols have been deposited, as well as through direct contact with the mucous membranes of the upper airway (generally the nose), as in the case of SARS-CoV-2 (3). In this respect, the best way to avoid exposure to aerosols is to prevent them from ejecting from the oral cavity. However, in the great majority of cases this is not possible, due to the duration of the treatments and the amounts of aerosols they generate (3).

In some cases, disinfection procedures are not entirely effective due to human error in applying them, or because in general, assessment of the efficacy of the procedure is based simply on visual inspection. This was evidenced in the study published by Whiteley *et al*. (1), where 74% of the surfaces analyzed after disinfection presented less than 100 colony-forming units (CFUs) – a measure that counts the number of viable microorganisms in a liquid or solid sample, and which determines the degree of microbiological contamination of a given environment.

On the other hand, the room air in the dental clinic may be contaminated not only from external but also from internal sources. The evidence suggests that the secondary contaminants, derived from oxidation of the primary contaminants - fundamentally volatile organic compounds (VOCs) - are the cause of the described adverse effects upon the health of patients and healthcare staff (9). In other words, the use of certain disinfectants could pose an added occupational risk. Hence the importance of choosing innocuous products, such as agents based on hydroxyl radicals (OH-), that protect the health of the professional.

In order for the disinfection process to be effective, we must take a number of factors into account, such as the optimum concentration of the disinfectant used, its application time, and the efficacy range. In this respect, the efficacy of disinfectants is defined by their antimicrobial spectrum (10).

The present systematic review was carried out to determine which methods for disinfecting the air and surfaces are most effective against the microorganisms present in healthcare environments, and to assess the current role in this respect of technology based on hydroxyl radicals (OH-).

## Material and Methods

The present systematic review was carried out following the preferred reporting items for systematic reviews and meta-analyses (PRISMA) statement (11) and it was registered in PROSPERO: CRD42021265224.

-Selection criteria

The key question raised in this study was: “Do hydroxyl radicals (OH-) achieve a greater decrease in microbial counts on surfaces and in room air compared with other disinfection methods?”

The review thus selected all those studies that included the items of the following PICO (patient, intervention, comparison, outcome) question:

- (P) Patient: dental clinic and/or hospital setting.

- (I) Intervention: use of hydroxyl radicals (OH.) as main disinfection method.

- (C) Comparison: comparison with at least one traditional disinfection method.

- (O) Outcome: the primary outcome variable was the bacteria colony-forming units (CFUs) on surfaces and in air. The secondary outcome variables were viral presence on surfaces and in air, and quantification of the volume of contaminated aerosols.

-Inclusion and exclusion criteria

We included “*in vitro*” studies on the disinfection methods used in the dental clinic and/or hospital setting, applied to both surface and room air.

Studies with missing data that could not be retrieved through contact with the authors were excluded.

-Search strategy

Two independent examiners (A.P-C and B.T-G) conducted the search in the Cochrane Library (Wiley), PubMed (MEDLINE) and Scopus databases in April 2022, with no restrictions in terms of language or publication date.

The following search strategy was used.

- PubMed: (“disinfection methods” OR “hydroxyl radical” [Mesh]) AND (“environment” [Mesh]) AND (“surfaces”) AND (“dentistry” [Mesh]).

- Cochrane Library and Scopus: (“disinfection methods” OR “hydroxyl radical”) AND (“environment”) AND (“surfaces”) AND (“dentistry”).

A search was also made in OpenGrey and www.greylit.org to identify grey literature. Likewise, ClinicalTrial.gov was explored to detect unpublished studies of relevance. Lastly, a manual search was made in the following journals specialized in the field: Journal of Clinical Biochemistry, Quintessence International, International Journal of Engineering Science, Indoor Air, Nutrition, and Journal of Hospital Infection, covering the period corresponding to the last 10 years.

-Selection of studies

The two independent examiners (A.P-C., B.T-G.) first selected the articles based on the inclusion criteria. A third reviewer (M.S-G) did not have to be consulted to resolve any discrepancies. Cohen’s kappa coefficient was used to assess agreement between the reviewers regarding the selected articles.

Based on the PRISMA statement (11), a first evaluation was made of the study title, followed by the abstract. Only those publications that met all the inclusion criteria subsequently underwent full-text evaluation.

-Data extraction

The information obtained from the articles was entered into Tables, with inclusion of the following data: author/s, year of publication, country, study design, evaluated disinfection method, and results of interest. In the case of any missing relevant information, the examiners contacted the authors of the publication.

-Risk of bias and quality assessment

A modification of the Cochrane risk of bias tool (RoB 2.0) was used to assess the methodological quality of the “*in vitro*” studies (12). Based on this, the evaluation focused on the following domains: randomization process, protocolization of the disinfection method, description of the environment to be disinfected (area, volume), bias in measurement of the results, and protocolization of the statistical analysis. The quality assessment was carried out independently by two authors (A.P-C and B.T-G) and, in the event of disagreement, consensus was reached through discussion with a third reviewer (M.S-G).

## Results

-Search findings

**Figure 1:**
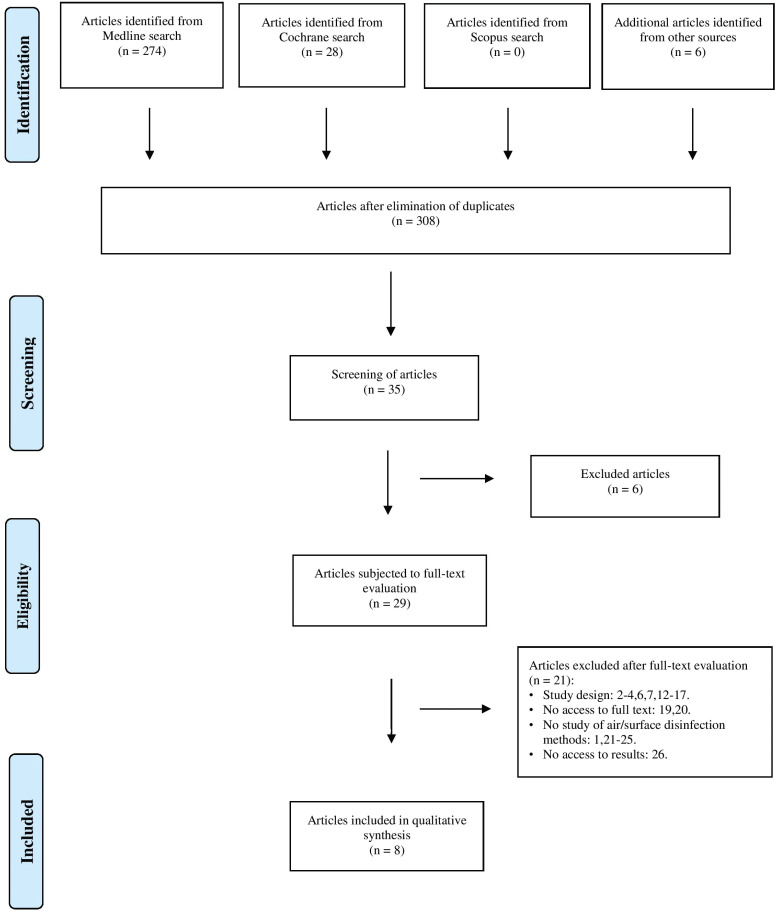
PRISMA statement flowchart.

The initial electronic search identified 308 articles. Following the removal of duplicates and of irrelevant articles based on the title and abstract, a total of 29 publications were subjected to full-text review. As can be seen in Figure 1, 20 articles were excluded due to the following reasons: 8 articles were excluded on the basis of their design (2-4,6,7,13-18), three due to the impossibility of full-text access (19,20), 5 because they did not investigate air and surface disinfection methods (1,21-25), and one because of the impossibility of accessing the results of the randomized clinical trial (26).

A total of 8 articles were finally included in the qualitative synthesis (9,27-33). Only three of them (29,30,33) were realized in hospital environment and no one in dental clinics. Inter-examiner agreement was excellent with a kappa coefficient of 1.

-Data extraction

-Qualitative synthesis

The 8 studies included in the review (9,27-33) were “*in vitro*” experimental studies. Two of them (28,33) used technology based on the release of OH- radicals. The study of Moccia *et al*. (29) evaluated an ozone (O3) releasing device, while Marchesi *et al*. (27) used a dry steam-based method. The studies of Moccia *et al*. (30) and Palcso *et al*. (31) evaluated different chemical surface disinfectants, specifically alcohol, chlorine, phenols, polyphenols, quaternary ammonium salts, tertiary amines, chlorhexidine gluconate and sodium chlorite (NaClO2).

Six of the included studies (28-33) evaluated the bactericidal effects of the disinfectants. The study of Martínez-Vimbert *et al*. (28) cultured Bacillus subtilis, *Staphylococcus aureus* and methicillin-resistant *Staphylococcus aureus* (MRSA), as well as *Pseudomonas aeruginosa*, *Salmonella*, *Klebsiella* and *Escherichia coli*. The study of Palcsó *et al*. (31) evaluated biocidal activity against *Enterococcus faecalis*, while Wong *et al*. (33) evaluated activity against bacteriophage MS-2 and *Staphylococcus epidermidis*. Lastly, Yamaguchi *et al*. (32) selected culture media for the growth of *Escherichia coli* and *Staphylococcus aureus*. Only two of the included studies (27,28) evaluated the effects of the disinfection methods in application to viral loads, specifically *Human Influenza* virus, *Respiratory Syncytial* virus (RSV), *Rotavirus*, *Echovirus* 7 and Coronavirus OC43 (HCoV). Only one of the studies (9) evaluated contaminating waste products (OHx and volatile organic compounds [VOCs]) generated after the application of a surface disinfectant based on glutaraldehyde and benzisothiazolinone and a hydroxyl radical (OH-) releasing device for the disinfection of room air (Table 1,  Table 1 cont.) show the methodological characteristics of the included studies.

**Table 1:**
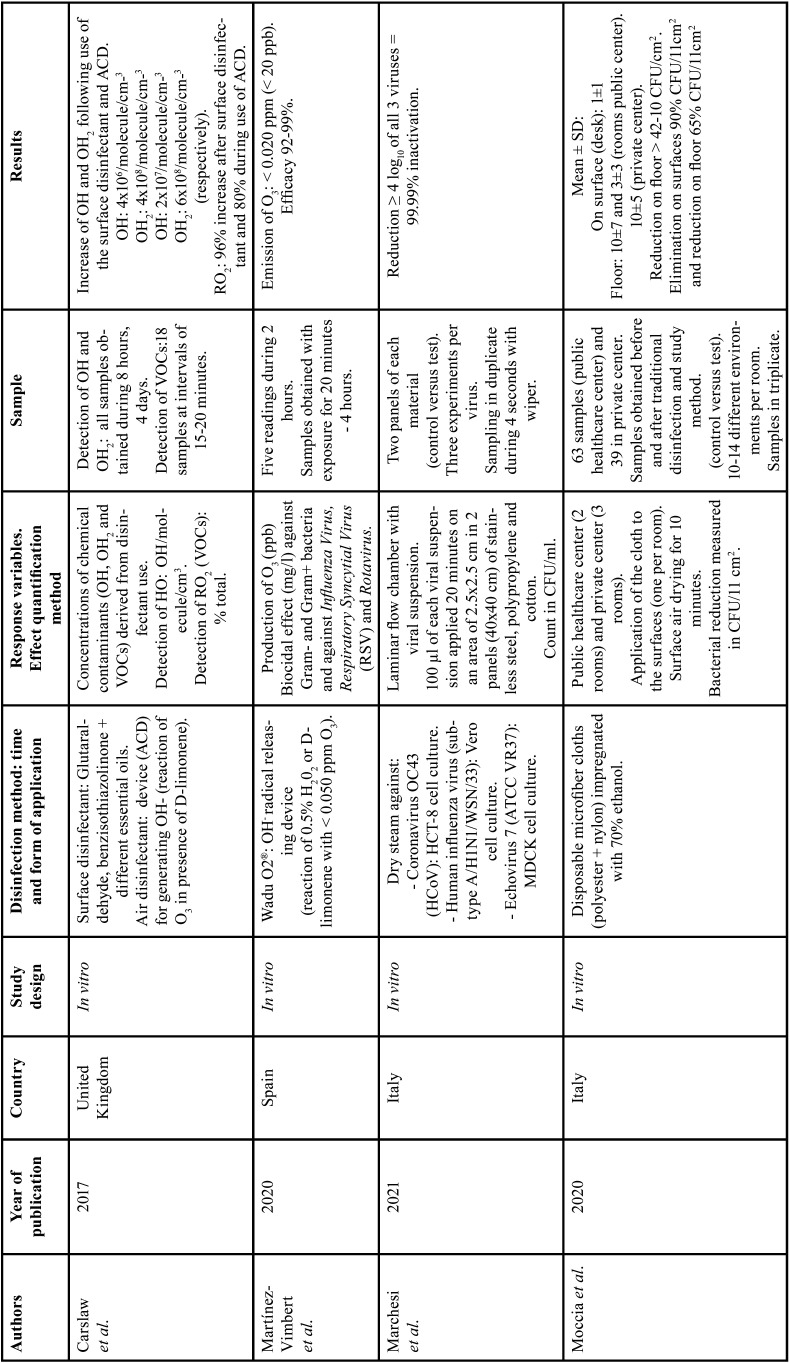
Studies included in the qualitative analysis: OH- (hydroxyl radicals), OH (hydroxyl), OH2 (hydroperoxyl), VOCs (volatile organic compounds), H202 (hydrogen peroxide), RO2 (peroxy radical), O3, (ozone), ppb (parts per billion), ppm (parts per million), HCT-8 cells (human epithelial cell line), MDCK cells (Madin-Darby kidney cells), CFU (colony forming units), Log10 (log units), µl (microliter), cm (centimeters), ml (milliliters), cm2 (cubic centimeter), NaClO2 (sodium chlorite), ClO2 (chlorine dioxide), m3 (cubic meter), CO2 (carbon dioxide), H2O (water),TSA (tryptic soy agar), TVC/m3 (total viable count per cubic meter), nm (nanometer), mW/cm2 (milliwatts per square centimeter), UV-A (ultraviolet radiation A), TiO2 NT (titanium dioxide nanotubes), ESR (electron spin resonance).

**Table 1:**
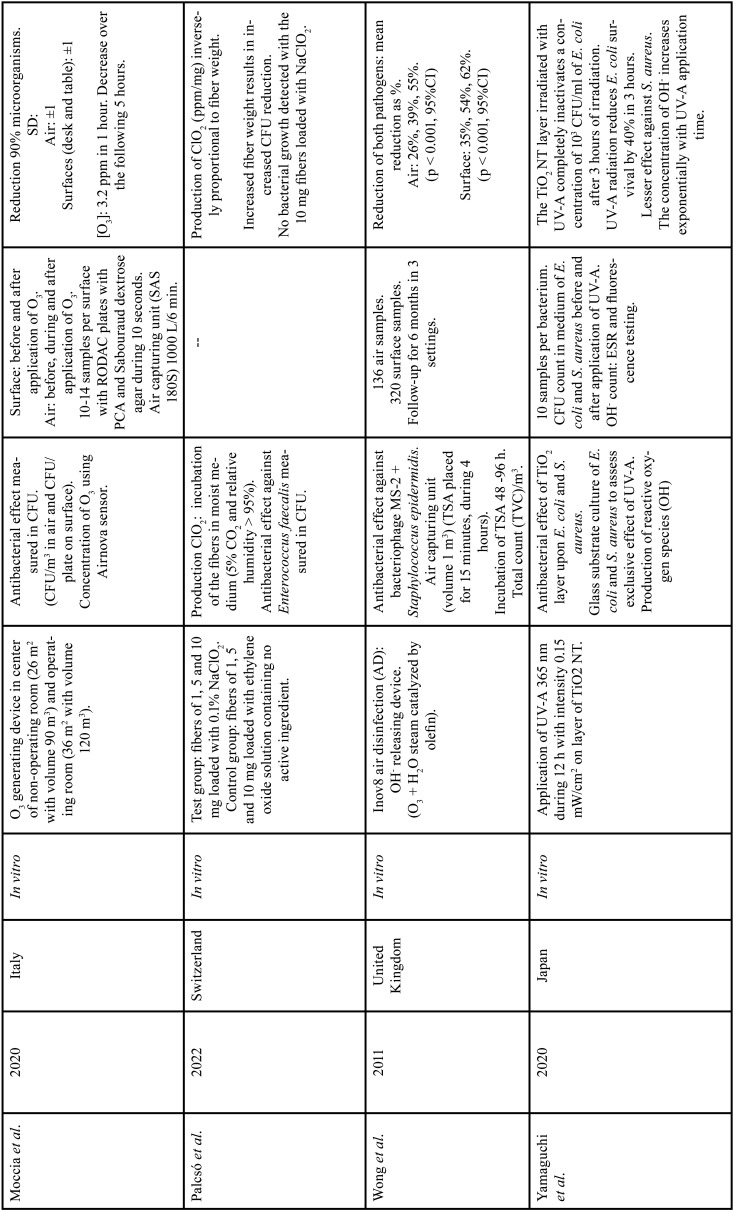
Studies included in the qualitative analysis: OH- (hydroxyl radicals), OH (hydroxyl), OH2 (hydroperoxyl), VOCs (volatile organic compounds), H202 (hydrogen peroxide), RO2 (peroxy radical), O3, (ozone), ppb (parts per billion), ppm (parts per million), HCT-8 cells (human epithelial cell line), MDCK cells (Madin-Darby kidney cells), CFU (colony forming units), Log10 (log units), µl (microliter), cm (centimeters), ml (milliliters), cm2 (cubic centimeter), NaClO2 (sodium chlorite), ClO2 (chlorine dioxide), m3 (cubic meter), CO2 (carbon dioxide), H2O (water),TSA (tryptic soy agar), TVC/m3 (total viable count per cubic meter), nm (nanometer), mW/cm2 (milliwatts per square centimeter), UV-A (ultraviolet radiation A), TiO2 NT (titanium dioxide nanotubes), ESR (electron spin resonance).

-Risk of bias and quality of the included studies

The methodological quality of the included “*in vitro*” studies was assessed using a modification of the Cochrane risk of bias tool (RoB 2.0) (12). The general quality of the studies was rated as low (Table 2). None of the “*in vitro*” studies designs involved randomization. One of the articles (9) failed to specify the concentration and time of application of the disinfectants used. Two of the studies (30,33) did not indicate the dimensions of the environments in which sampling was performed. Only three “*in vitro*” studies performed a quantitative statistical analysis of the data obtained (29,30,33).

**Table 2:**
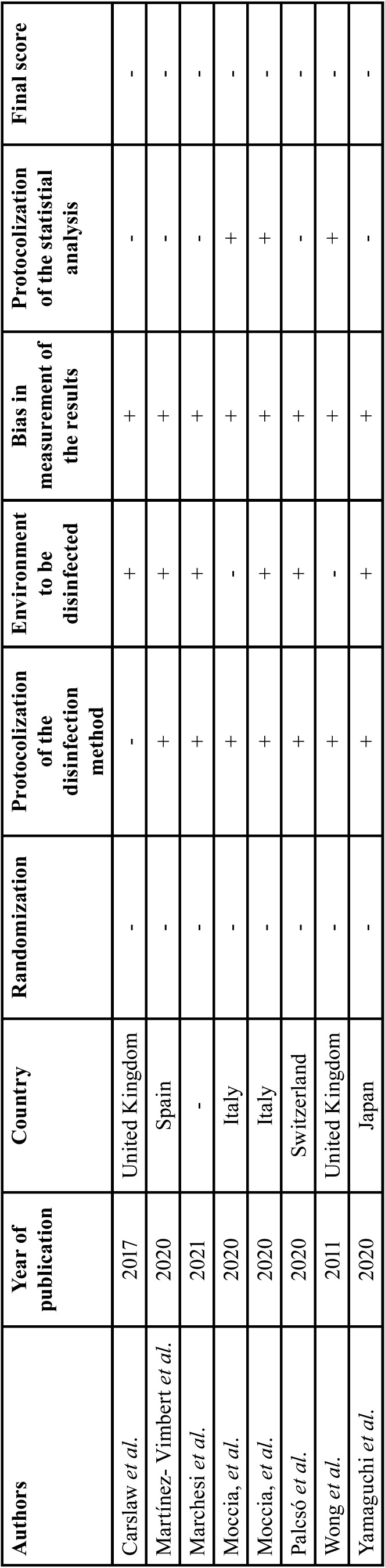
Risk of bias of the included studies. (+) low risk of bias; (-) high risk of bias.

## Discussion

The standard UNE-EN 13098:2019 (36), which also adopts the ISO international standard, determines the measurement of microorganisms and microbial compounds in suspension in the air by counting the number of microorganisms capable of growing and forming colonies in a solid medium following aerobic incubation at 30º.

-Disinfection of contaminated surfaces

According to the guide on the control of infections in dental practice published in 1993 by the United States Centers for Disease Control and Prevention (37), the surfaces in dental treatment settings are classified as critical, semicritical and non-critical, according to their need for disinfection or sterilization. Critical surfaces are those that need to be sterilized due to their high risk of infection, such as surgical instruments. Semicritical surfaces or objects include high or low speed rotary instruments that come into direct contact with the mucous membranes or skin. Those surfaces that do not come into contact with the skin in turn are classified as non-critical (38).

The need to achieve optimal disinfection of surfaces is evident in the study by Riddell *et al*. in which the presence of SARS-CoV-2 was detected for up to 28 days at 20 °C on surfaces such as glass and stainless steel (39).

Marchesi *et al*. (27) evaluated the effectiveness of dry steam against three viruses: coronavirus OC43 (HCoV) as a substitute of SARS-CoV-2, Human Influenza virus (subtype A/H1N1/WSN/33), and Echovirus 7 (ATCC VR37). The authors applied 100 µl of each viral suspension in a constant laminar flow chamber during 20 minutes. The investigators concluded that dry steam is effective in neutralizing the three viral species, with an inactivation rate of 99.99%, which corresponds to factor ≥ 4 log10.

The quaternary ammonium compounds, also known as QACs or quacs, are divided into 5 generations. These compounds exert their disinfectant effects by acting upon the enzymes, proteins and cell membrane (lipids) of the pathogen – fundamentally bacteria in the vegetative state and fungi. They afford high surface activity and can be used in combination with other disinfectants (17). Some of these disinfectants have been studied by Moccia *et al*. (30). These authors used disposable polyester and nylon cloths impregnated with different disinfectant solutions: 70% alcohol, 5% chlorine, 10% quaternary ammonium salts, 5% chlorhexidine gluconate and phenolic solutions that deactivate the enzyme system of the cell membrane, allowing the penetration of metabolites (17,30). The study concluded that all the chemical disinfectants were effective and were able to eliminate 90% of the surface CFU/11cm3. It should be noted that the biocidal effect on the floor was lower (only about 65% reduction of CFU/11cm3).

Another traditionally used method has been ozone (O3). However, its utilization has not been without some controversy, due to the possible risk of harmful effects for the healthcare staff and patients. In this regard, Moccia *et al*. (29) evaluated an O3 – generating device. The study was carried out in two hospital rooms: a non-surgical room and an operating room. The response variables were the antibacterial effect of the device upon the surface and air. It was concluded that O3 is able to reduce the presence of microorganisms in healthcare environments by up to 90%, with no added risk for human health, since the maximum concentration of ozone after the first hour of application was 3.2 ppm, and decreased exponentially over the following 5 hours (29). It is important to note that because of the associated risks, ozone exposure values are regulated. According to the National Institute for Occupational Safety and Health (40), the recommended O3 exposure limit is 0.1 ppm, which corresponds to 0.2 mg/m3. Only O3 levels ≥ 5 ppm are considered to pose an immediate health hazard (39). Regarding the use of ozone against the SARS-CoV-2 virus, there is insufficient scientific evidence regarding its efficacy and safety (41).

Chlorinated compounds have also been traditionally used. Palcsó *et al*. (31) evaluated sodium chlorite (NaClO2) impregnated in fibers of different sizes. The response variables were the production of chlorine dioxide (ClO2), which is the compound that really produces the biocidal effect, and antibacterial activity against Enterococcus faecalis. It was concluded that chlorine dioxide (ClO2), under conditions of relative humidity > 95% and 5% CO2, generated on impregnating the fibers with 1 and 5 mg of NaClO2, achieved a mean decrease in CFU/plate of 1.67 ± 2.87 and 1.00 ± 1.73 after 24 hours, respectively.

On the other hand, ultraviolet C radiation (UV-C) radiation has also been traditionally used in healthcare environments as a germicide for the disinfection of nosocomial pathogens in the air and in water. Its use is regulated by Specification UNE 0068, since wavelengths between 200-280 nm can have harmful effects for the skin of healthcare staff and patients (3,41). In this regard, ultraviolet radiation cannot be applied during healthcare working activities, in contrast to technology based on the release of hydroxyl radicals (OH-), which can be used without having to interrupt healthcare activities. Yamaguchi *et al*. (32) applied ultraviolet A radiation (UV-A) to a TiO2 NT surface against Escherichia coli (Gram-) and *Staphylococcus aureus* (Gram+). The authors concluded that UV-A radiation reduced the survival of Escherichia coli by up to 40% in the first three hours, since its impact upon the TiO2 NT surface gave rise to the formation of OH- radicals. The concentration of OH- increased exponentially with the radiation application time. The effect was less pronounced in the case of *Staphylococcus aureus*, due to the composition of its cell membrane. These morphological features cause this species to be less sensitive to the action of reactive oxygen species (ROS) such as OH- radicals. Regarding its efficacy against SARS-CoV-2, the available evidence is scarce and very heterogeneous (41).

-Disinfection of contaminated room air

Clarkson *et al*. (42) determined that aerosol generating dental procedures (AGP) are those that use high and low speed handpieces, air and water syringes, sonic and ultrasonic devices and surgical motors. Aerosols are differentiated according to particle size. Most aerosols produced in the dental clinic are under 5 µm in size (43). In this scenario, the use of physical protection barriers such as face screens is of great help, though such measures alone are unable to protect the dental professional and the patient from the inhalation of the smaller nuclei droplets (6).

Several techniques affording protection against aerosols have been described and are classified according to the timing of their application. On one hand there are techniques that prevent the contamination of aerosols generated within the oral cavity, while others prevent the projection of fomites outside the oral cavity. In turn, ventilation techniques are intended to avoid aerosol spread beyond the operating zone, while other general ventilatory methods aim to prevent particles from spreading outside the dental office. Direct decontamination of the aerosol corresponds to the last phase of the disinfection process (6).

The first protective barrier against aerosols consists of controlling the endogenous microbiota of the oral cavity of the patient (6,43). Emerging studies demonstrate the importance of the oropharynx and oral salivary glands as sites of replication and transmission of microorganisms. In a study conducted by O’Donnell *et al*. (44) was evaluated the action of mouthwashes, such as chlorhexidine or cetylpyridine chloride (CPC), on the disruption of the lipid membrane of viruses such as SARS-CoV-2. These mouth rinses have been shown to potentially reduce the transmission of SARS-CoV-2 and therefore a mouth rinse prior to dental treatment is recommended to reduce oral microbial load. Also in the study by Meyers *et al*. (45) mouthwashes were shown to be up to 99.9% effective in inactivating human coronavirus (HCoV) with a contact time of only 30 seconds. In recent years, cetylpyridinium chloride (CPC) has been incorporated to use in the dental clinic. This is a cationic quaternary ammonium compound with great antiseptic capacity, as evidenced by the randomized clinical study published by Maximo *et al*. (46) in 2020, in which three test groups (0.12% chlorhexidine, essential oils and 0.07% CPC) were compared against a control group with 0.5% water-alcohol solution. The authors concluded that CPC achieved the greatest decrease in bacteria in patients with periodontal diseases.

Conventional saliva ejectors are essential for preventing particles from escaping from the oral cavity. However, such low-volume aspirators are generally unable to neutralize all the generated aerosols. In this respect, high-volume evacuation (HVE) systems are needed to prevent aerosol dispersion beyond the surgical zone. These systems are able to evacuate up to 2.8 m3 of air per minute, reducing aerosol presence by up to 90.8% (6). In this regard, Wan Hassan *et al*. (26) are currently conducting a parallel-group randomized clinical trial to evaluate the processing of aerosols generated during dental treatments, in order to avoid cross-infections, comparing the conventional aspiration system of a dental clinic versus a new high-volume evacuation system. Once aerosols escape from the surgical zone and are suspended in the environment of the dental office, the general ventilation system is responsible for neutralizing them. It is important to avoid the use of ventilating fans, since they facilitate recirculation of the contaminated air, maintaining correct ventilation of the dental office by opening the windows. However, and above all, it is necessary to install a high-performance filtering system, involving the use of HEPA filters or high-efficiency particle filters (6).

-Technology based on the release of hydroxyl radicals (OH.)

In view of the need to develop a rapid and effective method for protecting healthcare environments from cross-infections, an environmental method has been developed for the elimination of pathogenic microorganisms in large spaces and surfaces. This technology is based on the release of reactive oxygen species (ROS) in the form of hydroxyl radicals (OH-). The OH- radical is the most important natural oxidant in the troposphere, and plays a key role in the elimination of greenhouse effect gases (28). The biocidal actions of OH- radicals begin through solar radiation, with the degradation of organic compounds transported in the air into harmless organic compounds. Such radicals are therefore included within the “green oxidant” concept, since they decompose into water (H2O) and oxygen (O2). Their function is mediated by an advanced oxidation process (AOP) that takes place in the membranes, lipids and hydrosulfide bonds of the proteins and nucleotides of DNA, giving rise to lipid peroxidation, cross-bonding between proteins, and mutations of the genetic material of pathogenic microorganisms (28). This technology affords several advantages with respect to the traditional chemical disinfectants such as chlorinated agents or quaternary ammonium compounds. Firstly, OH- radicals are not selective and can eliminate any pathogen with very low doses (0.8 mg/l), with an oxidation potential of 2.8 vatios (V). In addition, their processing time is very short (4 seconds), since their reaction velocity is high and constant, specifically 109 L/mol/sec and they have a persion-dispersion density of 22 ml/cm2, equivalent to a thousandth of that of other disinfectants (28). Since technology based on OH- release produces oxidative damage to lipid membranes, with denaturalization of proteins and the modification of nucleic acids of pathogens, its utilization may have adverse side effects.

In this regard, Carslaw *et al*. (9) evaluated the production of hydroxyl radicals (OHX) generated following the use of a surface disinfectant composed of glutaraldehyde, benzisothiazolinone and a number of essential oils, and with the utilization of an air disinfection device. The first measurements indicated an initial hydroxyl radical and hydroperoxyl (hydrogen superoxide) radical (OH2) concentration of 6.5 x 105 cm-3 and 1.3 x 107 cm-3, respectively. After disinfection of the surfaces, the concentrations of hydroxyl radicals and OH2 increased to 4 x 106 cm-3 and 4 x 108 cm-3, respectively. In turn, following activation of the air disinfection device, the concentrations reached 2 x 107 cm-3 and 6 x 108 cm-3.

An analysis was also made of the production of potentially contaminating secondary chemical species, specifically peroxyl radicals (RO2), which are generated following the oxidation of volatile organic compounds (VOCs). An increase in RO2 of between 80-96% was recorded, derived mainly from two types of VOCs: terpenes and aromatic groups. In addition, the production of RO2 was seen to be greater after applying the chemical surface disinfectant versus the air purifying device. This implies two important things: on one hand, the bactericidal effect of the disinfectants is mediated by highly reactive chemical mechanisms that can also produce secondary chemical agents, and on the other, the disinfectant application medium is a fundamental factor – not only to ensure greater bactericidal effects but also to reduce the production of potentially contaminating secondary compounds (9).

In contrast, Martínez-Vimbert *et al*. (28) concluded that the use of an OH- releasing device is entirely safe and innocuous. These authors conducted different laboratory studies with two reactive agents: hydrogen peroxide (H2O2) and D-limonene (a natural terpene). The hydrogen peroxide output with the studied device was 0.9 mg/m3, which is equivalent to 0.64 ppm, a concentration lower than that considered to be toxic for the respiratory tract. The natural terpene D-limonene has double carbon bonds, allowing it to interact with O3, generating OH. radicals and other sTable products such as ketones or carboxylic acid. It is postulated that the potential harmful effect of D-limonene is due to the fact that this reaction can also generate volatile organic compounds (VOCs), generally glutaraldehyde. However, this is not harmful, due to several reasons. Firstly, the production of D-limonene is 1.84 parts per million (ppm) - a concentration that is lower than the limits established by the regulations of other European countries such as Sweden, with a limit of 27 ppm, or Germany, with 10 ppm. Secondly, its evaporation is below 2 ppb (parts per billion) in a space of 60 m2. Furthermore, the emission of O3 is less than 0.02 ppm.

Another issue related to this technology is its utilization in large spaces. In this regard, Wong *et al*. (33) carried out an “*in vitro*” study using an OH- releasing device (Inov8®, Buckingham UK) in different areas of a hospital center. The OH- radicals were generated as a result of the reaction of O3 with water vapor (H2O), catalyzed by a terpene (D-limonene). The results were satisfactory, since there was a decrease in bacteriophage MS-2, composed of single-strand RNA, and in *Staphylococcus epidermidis*, a facultative anaerobic Gram+ bacterium. Specifically, the reductions were between 26-55% in air samples and between 35-62% on TSA (Tryptic Soy Agar) plates for total aerobic bacteria, respectively.
